# Bridging cultural sensitivity and ethical practice: Expert narratives on child and adolescent mental health in Kuwait

**DOI:** 10.1177/13591045251380307

**Published:** 2025-09-24

**Authors:** Kaouthar Chebli, Alice Gritti, Clara Calia

**Affiliations:** 1 3124 The University of Edinburgh, UK; 2 School of Health in Social Science, 3124 The University of Edinburgh, UK

**Keywords:** Mental health, cultural factors, stigma, family, religion, children, Kuwait

## Abstract

**Aims:**

This study examines the influence of cultural factors on mental health perceptions and practices in Kuwait, with a specific focus on mental health professionals working with children and adolescents. Employing the ETHICA-4P toolkit, the study aims to evaluate and promote ethical and culturally sensitive approaches in research and clinical interventions.

**Methods:**

A qualitative research design was used, involving semi-structured interviews with mental health professionals to explore their experiences and approaches to culturally sensitive practice.

**Results:**

Findings indicate that cultural factors, such as family dynamics, religious beliefs, and societal stigma, strongly influence help-seeking behaviours and therapeutic outcomes. Despite available mental health services, cultural stigma and fear of social repercussions remain significant barriers in Kuwait, including concerns about family reputation, judgment from the community, and potential impacts on marriage prospects or social standing.

**Conclusion:**

ETHICA-4P can act as a model to integrate cultural competence into clinical practice and improve mental health outcomes for children and youth. Findings also highlight the urgent need for education campaigns directed at therapists, parents, educators, and the general public to reduce stigma and increase mental health awareness.

## Plain Language Summary

Mental health challenges among children and teenagers are growing around the world, including in Kuwait. Therapists working with children and adolescents often face difficult situations where they must balance what is best for the young person with what is expected by families and society. These situations can involve sensitive topics like family conflict, religion, or stigma around mental health. This study looked at how therapists in Kuwait think through such ethical challenges when supporting adolescents. We interviewed a small group of Arab therapists who work with children and teenagers in Kuwait. They shared stories from their clinical work and reflected on how they make ethical decisions when the right answer is not always clear. We also asked them about a new tool called ETHICA-4P, which is designed to help therapists think carefully about ethics in different cultural contexts. The therapists explained how their own cultural understanding helped them build trust with children, teens, and families. Some described how they used creativity to adapt their approaches—like involving parents in new ways or finding ways to protect a child’s privacy without harming family relationships. While the ETHICA-4P tool was seen as helpful in organizing their thoughts, therapists also said it would need to be adapted to better fit the Kuwaiti context. This study helps us understand the real-life decisions therapists make in culturally complex environments. It shows the importance of cultural sensitivity, professional experience, and flexibility when supporting adolescent mental health. It also suggests that tools like ETHICA-4P can be useful, but only if they are adjusted to reflect local values and practices. Future research could look at how such tools can be refined and used more widely across different cultures.

## Introduction

Understanding the cultural nuances of Kuwaiti society is particularly important for therapists working with clients from diverse backgrounds. Kuwait is divided into six health regions, each functioning as a decentralized administrative unit. Each region is served by a general hospital, multiple Primary Health Care (PHC) centres, and specialized clinics. The Kuwait Centre for Mental Health, located in Al-Sabah health region, serves as the Ministry of Health’s (MOH) primary psychological and psychiatric care facility, offering specialized services for both inpatient and outpatient treatment of adults, children, and adolescents. Across Kuwait, the MOH operates 115 PHC public clinics as well as a growing number of private sector clinics, some of which include specialized mental health services (UNICEF, 2023).

In the Arabian Peninsula, mental health issues are often viewed through religious and traditional lenses (Gearing et al., 2013a, 2013b). Kuwaiti culture prioritizes maintaining family and clan reputation, over personal matters (Kuwait Society & Culture, 2010). Individuals act carefully to avoid undermining their family’s honour, dignity, or social standing. This dynamic creates a burden in managing sensitive issues, for example , an individual will want to deal with mental ill-heath privately instead of seeking professional help (Elshamy et al., 2023). Concerns about family reputation, fear of being labelled “sick,” and doubts about therapy’s efficacy contribute to this reluctance (Almazeedi & Alsuwaidan, 2014; Khatib et al., 2023). Many individuals turn to general practitioners instead of mental health clinicians due to stigma, fearing repercussions on relationships, self-perception, and marriage prospects (Almazeedi & Alsuwaidan, 2014; UNICEF, 2023). Recent efforts have increased mental health service integration within primary healthcare through public-private collaborations.

Adopting a cultural lens is essential for effective mental health support, as it informs both therapeutic outcomes and client expectations (Chandra et al., 2016). In June 2024, the Public Authority for Civil Information (PACI) reported Kuwait’s population as 1.5 million Kuwaitis and 3.3 million non-Kuwaitis, comprising Asians (40.3%), Arabs (27.4%), and Africans (1%) (Kuwait Times, 2024). The largest migrant communities include Egyptians, Indians, Filipinos, Bangladeshis, and Syrians (Toumi & Chief, 2016). Understanding these cultural attitudes toward help-seeking —across both Kuwaiti and non-Kuwaiti communities— is crucial for therapists to build rapport and deliver effective interventions. Throughout this study, the term “population” refers to the adolescent clients receiving therapy in Kuwait. This includes both Kuwaiti nationals and non-Kuwaiti residents, reflecting the multicultural composition of the country. In the interviews, therapists were invited to reflect on their clinical work more broadly, and while many examples related to Kuwaiti clients, their responses also addressed experiences with adolescents from various cultural backgrounds residing in Kuwait. This nuance is important in interpreting the themes presented in the findings.

In this study, culture is understood as a dynamic and evolving system of shared values, beliefs, and practices, that shape how individuals and communities interpret and respond to life experiences. Within the Kuwaiti context, it is important to distinguish between the dominant cultural norms held by Kuwaiti nationals, specifically, as well as Arabs, generally—often influenced by religion, family roles, and tradition—and the broader multicultural fabric of the country. Acknowledging this complexity allows for a more precise critique of specific cultural dynamics that may present challenges in mental health care, without framing culture itself as a problem. Instead, this approach emphasizes the need to critically engage with the cultural assumptions embedded in practice, in order to improve therapeutic outcomes and ensure interventions are both respectful and effective within this unique socio-cultural landscape.

**Figure 1.**
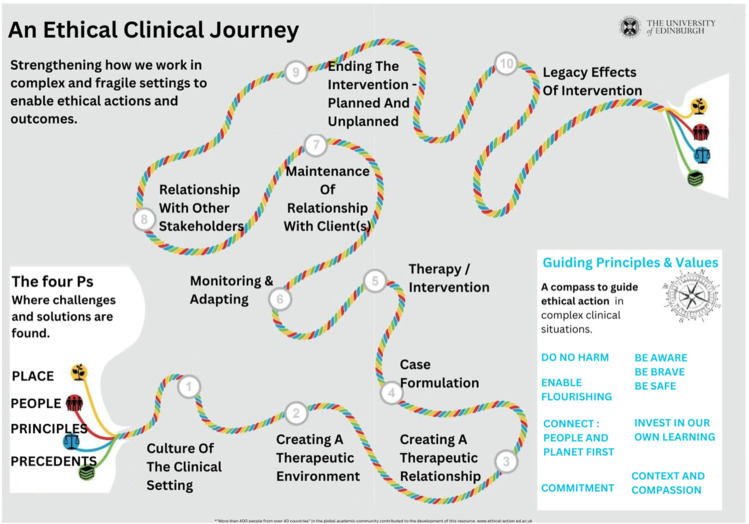
Ethics in clinical practice, ETHICA- 4P https://www.ethical-action.ed.ac.uk/clinical-practice.

Despite the growing need for culturally informed mental health services, Kuwait lacks a specific ethical framework addressing stigma and complex intercultural clinical challenges (Merhej, 2019). It remains uncertain whether tools adapted to the Kuwaiti context —including both Kuwaiti nationals and the diverse expatriate communities— exist to operationalize general ethical principles effectively. To address such gaps, international frameworks, such as ETHICA-4P (Figure 1), offer valuable guidance (Reid et al., 2019). Designed for ethical clinical practices in intercultural settings, ETHICA-4P emphasizes bridging cultural differences while adhering to ethical standards. This makes it particularly useful for practitioners working in culturally diverse environments like Kuwait, and more broadly, in other countries with similarly multicultural populations.

The toolkit outlines ten steps across the clinical journey, including understanding the clinical setting’s culture, fostering a therapeutic relationship, case formulation, intervention, monitoring, and closure, with a focus on legacy effects. ETHICA-4P is anchored in four guiding influences—Place, People, Principles, and Precedents—highlighting the significance of local context, cultural values, professional regulations, and established practices in navigating ethical and clinical challenges. By integrating these considerations, ETHICA-4P provides a structured approach to culturally sensitive and ethically sound mental health interventions.

This study explored the challenges faced by child and youth mental health clinicians in Kuwait as they work to address and reduce the stigma associated with mental health in their clinical practice. ETHICA-4P was utilized in this study to investigate its applicability in the clinical field for children and young people in Kuwait. Additionally, it highlighted the clinicians’ readiness for cultural adaptability (Sewilam et al., 2015) in conceptualizing cases and designing interventions and the potential ethical or cultural challenges they might experience.

## Methods

### Data gathering

Purposive sampling was utilized to recruit mental health professionals from both private and public sectors in Kuwait, specializing in child and adolescent care. Of 22 individuals contacted, seven consented to participate. Inclusion criteria required participants to have a minimum of two years of professional experience in mental health with children and adolescents (as well as an initial meeting/ interview with their parents) and to demonstrate familiarity with Kuwaiti cultural contexts. Participants in this study were mental health professionals providing therapeutic services to adolescents in Kuwait. At the time of the interviews, therapists were engaged in a range of modalities, including both individual therapy with adolescents and family-based interventions. The choice of modality was determined by clinical need and organisational setting. This diversity in practice allowed for a broader exploration of how therapists approached adolescent mental health within different relational and cultural dynamics. All participants self-identified as Arab and were culturally familiar with the Arab and Kuwaiti sociocultural context. This cultural alignment was considered important given the study’s focus on culturally embedded clinical experiences in Kuwait.

The study focused on exploring therapeutic approaches, ethical challenges, and culturally tailored interventions to assess the impact of cultural sensitivity on mental health outcomes for this population. The ETHICA-4P framework was introduced in this study as an exploratory tool to guide discussions around ethical practice in culturally diverse settings. Chosen for its emphasis on ethical reasoning across intercultural contexts, the framework served as a point of reflection for participants to evaluate its practical relevance in Kuwait. While the model provided a structured lens to assess ethical challenges, it has not yet been validated within the Kuwaiti context. This study does not attempt to promote ETHICA-4P as a universal solution, but rather initiates a critical dialogue on its perceived applicability, cultural fit, and potential for adaptation.

All interviews were transcribed using the online transcription tool *Otter* (https://otter.ai/home), after which transcripts were thoroughly proofread to ensure accuracy and fidelity to participants’ responses. The qualitative data analysis software NVivo 14 (Lumivero, 2023) was then employed to organize the narrative data, initially categorizing it into multiple codes, from which themes were subsequently generated.

### Data analysis

Reflexive thematic analysis (Braun & Clarke, 2006) was employed using a deductive approach to explore how culture influences therapy, focusing on Kuwait’s mental health context to improve case conceptualization and intervention design (Braun and Clarke, 2013, 2019, 2021). Pseudonyms were used to ensure confidentiality. The use of reflexive thematic analysis allowed for a nuanced exploration of how participants make meaning of cultural and clinical complexity. While the findings are context-specific, they highlight recurring themes that may inform future research and culturally responsive practice.

As an interviewer with an Arab background, cultural familiarity with Kuwait’s norms and mental health stigmas facilitated respectful, open dialogue. This cultural insight enhanced data quality by enabling nuanced interpretation of participants’ responses, integrating both cultural awareness and professional understanding.

## Results

### Participants

Most participants held qualifications in psychology, educational psychology, or mental health counselling (Table 1).

**Table 1.** Socio-demographic characteristics of participants.

Participant	Gender	Profession	Sector	Years of experience
Ahmad	M	Neuropsychologist	Private	11
Amal	F	Clinical psychologist	Private	6
Leila	F	Psychoeducational therapist	Private	25
Nour	F	Psychologist	Public	4
Najla	F	Educational psychologist	Private	15
Fawzia	F	Mental health counsellor	Private	4
Sarah	F	Psychologist	Public	2

Looking at mental health from a cross-cultural context, three primary themes were identified from the participants’ narratives: (1) cultural influences on mental health perception, (2) trust in culturally sensitive care, and (3) actions to shift the stigmatized mental health narrative Subthemes were also identified, offering valuable insights that can inform targeted interventions (Table 2).

**Table 2.** Themes and Subthemes.

Themes	Subthemes
Cultural influences on mental health perception	The therapeutic relationship and opposing cultural beliefs Family Religion
Trust in culturally sensitive care	Cultural context of trust Overcoming cultural barriers to trust
Actions to shift the stigmatized mental health narrative	Parent coaching ETHICA-4P

### Cultural influences on mental health perception

#### The therapeutic relationship and opposing cultural beliefs

Participants frequently noted that the cultural backgrounds of both the client and therapist influence the session’s structure and the intervention design. Ahmad mentioned, “I understand that this is the background of my patient. This will allow me to understand their way of thinking and dealing with issues”. Understanding the client’s background can guide how effectively the session will be structured and conducted. In this context, incorporating cultural adaptation into mental health therapy enables clinicians to align their methods with the unique cultural contexts of their clients, thereby enhancing mutual understanding and rapport. By integrating clients’ cultural beliefs, values, and practices, therapists can offer more effective and respectful care, leading to improved therapeutic outcomes and client satisfaction. Ahmad also highlighted the importance of exploring the client’s history and the events that may have impacted their mental health.

Amal said that “clients might choose me because they see me as someone who understands their cultural context and won’t impose foreign values on their children”. This is an example of how clients, especially parents, would in most cases prefer a therapist with a similar cultural background to theirs so that expectations and influences align. Amal added that parents sometimes choose her as their child’s therapist because, as an Arab, she shares a similar cultural background to those in Kuwait, being family-oriented, familiar with comparable family structures, and understanding social expectations for girls and boys. However, other times clients might prefer a therapist from a different cultural background, that is; from a different cultural and belief system (Westerners). Leila provided an example of an Arab client who did not want to be assessed by an Arab professional:

What was triggering her from the beginning before she started the assessment was when she came in and when she discovered that I'm not American or European. I could see the shock on her face. She told me I came here, and I thought that you are foreigner. Oh, when I discovered that you are Arab, I don't like Arab people. I don't want you. I don't want to open up for anyone. I want someone American. (Leila)

Leila explained “Some people feel more comfortable with European or American because they, you know, in our Arabic culture, there are some people they don’t trust. They think that we are going and tell their issues or something”. This quote illustrates the importance of cultural awareness in therapy, especially when the therapist faces diverse perspectives that can complicate the process of establishing a common approach to addressing specific issues.

This challenge in building a therapeutic relationship usually occurs when cultural/religious values and personal opinions clash. Nour mentioned how some parents might disagree with the treatment approach, which might lead to discontinuation of the session or referral to other therapists; “sometimes parents have unrealistic expectations or their opinions clash with mine. For example, if there’s a significant disagreement with the parents about the treatment approach, I discontinue the sessions”. She also added how sometimes parents would interfere during the sessions themselves, questioning her choices. Overall, participants consistently emphasized the need to raise awareness about mental health perceptions and practices in Kuwait, starting at the individual level with parents, teachers, and families, and extending to the governmental level, reaching schools and mental health clinics.

#### Family

Ahmad mentioned that since the family can sometimes get in the way of the therapeutic relationship or could cause stress and anxiety in the children, it is important to learn how to set boundaries so that the client does not lose a sense of belonging in such a sensitive surrounding.

In the GCC (Gulf Cooperation Council) countries, the family has a major role in everything, in their major choices like marriage and number of kids and whatever. I always try to work on with my patients in the GCC to reconnect with the family, but in a healthy way, which means they have to learn how to set boundaries. (Ahmad)

Najla talked about one of her adolescent clients whose mother was always concerned about the opinions of extended family members. Her father “made her do so many things that she wasn’t completely comfortable with, like wearing hijab (headcover)”. After he passed away,

She said that I don't feel comfortable wearing hijab, it's not me, so I have to remove it. But her mom wasn't very supportive. So, she told her, you still have to wear it when you're with family. Sometimes she has to wear it, sometimes she doesn't. She feels guilty when she doesn't. (Najla)

This reflects how individual boundaries can be overlooked in comparison to familial boundaries.

Stigmatizing mental health conditions leads to future burdens, especially where family reputation outweighs individual experiences. Leila provided an example of parents feeling offended when given a referral. She recounted an incident where a mother sought support for her child’s inappropriate behaviour stemming from family conflicts. Upon advising the mother to consider family therapy, the father reacted aggressively, talking to the school principal about Leila, “She shouldn’t say this to my wife, why do we need family therapy? And please tell her that I can wait for her outside of the school to harm her.” The father’s reaction could be traced back to imposed notions of stigma and privacy, commonly observed in Arab cultures where family reputation takes precedence over individual needs (Al-Krenawi & Graham, 2000; Coker, 2005). Such cultural dynamics are rooted in maintaining honour and avoiding shame, which influences attitudes toward mental health help-seeking (Gearing et al., 2013a, 2013b; Sewilam et al., 2015).

Interviewees described other challenges experienced during the therapeutic process. According to Fawzia, “Kuwait is small, everybody knows everybody. And like, I’ve had situations where I’m meeting the kids, and then the parents come in, and we’re somewhat related, or we’re related through marriage. Some of the times I do struggle with that”. This quote emphasizes how Kuwait’s tight-knit community often reveals unexpected family connections, which can create challenges in personal and therapeutic relationships.

#### Religion

Religion was also brought up in the interviews, especially since mental health could sometimes be intertwined with religious contexts.

Sometimes religion might affect the way I intervene. It might look kind of weird to mention religion, but religion has a very, very important impact on people, way of thinking and behaving and dealing with issues (Ahmad).

According to Halaj and Huppert (2017), mental health disorders are sometimes attributed to religious concepts like spiritual forces, envy, and sorcery, with terms like “waswas” (referring to insinuating whispers from the Devil) reflecting both psychological and religious contexts. Although religion is relatively seen as supportive of mental health and overall well-being (Schumaker, 2023), it is sometimes seen as an influence of mental “illness” in the Muslim practicing community due to “lack of faith or disconnection from religion” (Alyafei et al. 2021, p. 18, 2021). This was brought up by Sarah who said:

Me personally, on my family level, I faced a difficulty at the beginning to make them understand that I'm really doing something big. Especially in our Islamic society, although it has nothing to do with religion in the first place. “Pray and read the holy book and you will be fine”, “if you go to a therapist, you are crossing the boundaries in religion, why are you going to someone other than God ‘Allah’”.

These are some of the narratives young individuals in need often would deal with, whilst trying to make sense of the world around, religion included. Amal stated how some parents would want to have their children see her solely because she wears the hijab (headscarf) and that would be close to them and their upbringing. As a response to that, she said; “I set clear boundaries and explain my role in helping their child. I am not here to impose my values or to act as a religious guide”. In doing so, she maintains professionalism while ensuring her role is understood and respected.

### Trust in culturally sensitive care

Trust emerged as a significant theme in understanding the dynamics of culturally sensitive mental health interventions for children and young people in Kuwait. The cultural fabric of Kuwaiti society, deeply rooted in religious and traditional values, influences how mental health issues are perceived and addressed. Building trust between mental health professionals, young clients, and their families is both a challenge and a necessity in providing effective care.

#### Cultural context of trust

In Kuwaiti society, the role of family and community is paramount. Families are often the gatekeepers of young people’s access to mental health services, and their trust in the therapist plays a crucial role.

Several years back, I took a promise on myself that I will never see parents alone, and whatever they want to say they will say it in front of their kids, so the kids gain trust because trust is the main issue. (Najla)

This emphasizes the importance of fostering trust within the therapeutic relationship, ensuring transparency and strengthening the bond between parents, children, and the therapist. Sarah also mentioned that “I gather a complete history from the family. We always assess the educational level, occupation, and living conditions of the family during the initial evaluation. This information can reveal cultural and socio-economic factors that might affect the child.” This underscores therapists’ need to demonstrate cultural competence and sensitivity, ensuring that interventions align with the family’s expectations and cultural norms.

#### Overcoming cultural barriers to trust

In Kuwaiti society, stigma surrounding mental health and fear of judgment often undermine trust in therapeutic settings. Families may worry about the social consequences of seeking help, particularly in matters related to marriage or public reputation. Amal noted, *“Parents sometimes try to persuade me to continue without medication, fearing it will open a file or involve identity verification.”* Especially for those approaching adulthood, having a medical record that includes engagement in mental health treatment, could affect future decisions when it comes to employment or marriage, particularly for women. These records may become known through work or family marriage inquiries, as connections and social networks often lead to shared information, fuelling stigma and influencing decisions. These concerns reflect broader cultural anxieties, where maintaining family honour can outweigh the importance of addressing mental health issues. Religion also plays a pivotal role in shaping trust. While religious beliefs can offer comfort, they can also create barriers when mental health struggles are framed as a lack of faith or moral failing. Sarah shared that “pray and read the holy book and you will be fine” would be circulating between her family members. Therapists must navigate these beliefs sensitively, ensuring that their interventions align with cultural and religious values while addressing the underlying issues.

The cultural background of the therapist significantly influences trust dynamics. Some families prefer therapists who share their cultural context, believing they are better equipped to understand and respect their values. Amal shared, *“parents sometimes choose me because they see me as someone who understands their cultural context and won’t impose foreign values on their children”*. However, other clients may prefer therapists from outside the region, fearing that local professionals might compromise confidentiality or judge their behaviour as Leila’s experienced with one of her clients who didn’t want to be assessed by an Arab professional. By prioritizing trust, therapists can create a safe space where children and adolescents feel supported in addressing their mental health challenges. This approach not only enhances the effectiveness of interventions but also helps to shift societal attitudes, reducing stigma, and fostering greater acceptance of mental health care in Kuwait.

### Transformative approaches to mental health narratives

Participants shared a common hope to reshape the mental health narrative in Kuwait, particularly to support parents and their children facing challenges in accessing resources while coping with societal judgments, stereotypes, and stigma. Parent coaching and the transparent therapeutic relationship were a few positive actions gathered from the interviews.

#### Parent coaching

Participants emphasized the need to raise awareness and educate parents on mental health, feeling the responsibility to encourage parental cooperation in addressing these issues. Fawzia said, “A big part of what I do is educating the parents, especially when the kids are younger. Parent education is very, very vital. I feel having separate sessions, just for parents.” Ahmad also mentioned that parents “need to be coached,” and when it comes to designing the intervention, “I always make sure that whatever I’m implementing, parents are okay with that, because they have to repeat it”.

Najla emphasized the importance of coaching parents and letting them fully express how they feel in front of their kids; “whatever they want to say they will say it in front of their kids, so the kids gain trust”. Building trust between parents and children involves setting boundaries, particularly when it comes to mental health services.

Parents are often confused and don't know how to deal with many issues, so they don't come back. I also educate parents on their role and what they need to do. Some parents understand and follow the guidelines, while others struggle due to their own issues and stress. (Amal)

Leila highlighted the importance of acknowledging that parents are human and may need guidance on supporting their child/ren in need, “I take into consideration a lot, their sensitivity. I don’t blame them. I don’t let them feel guilty. I try to listen to them. So, I expect their emotions. Sometimes they need therapy.”

#### ETHICA-4P

Participants emphasized the importance of having ETHICA-4P in their practice. Sarah mentioned how having such a guideline makes it easier for practitioners to have a reference point whenever needed; “It is clear and easy to remember. It makes sense. It is very smooth to follow”. Having a tool that is easily accessible for practitioners helps improve the quality of the session as well as the therapeutic relationship. Nour mentioned how “the model is very comprehensive and aligns with what we are supposed to do.” She also added how essential it is to gather the complete history of not just the client, but the family as well considering that she works with children and adolescents. She also referred to gathering the history of some extended family members who could have a direct impact on the client as well. Another positive feedback of the ETHICA-4P was that:

Overall, I do feel it has a good flow. The monitoring and adapting phase for sure with kids is very, very crucial. Just to see how they're doing, how they're flourishing, as they grow older, so yeah, I do believe it's a very good, interesting model. (Fawzia)

The steps of ETHICA-4P are the main steps in the majority of the clinical therapeutic approaches. However, the application of these steps is unique because it is focused on diversity. This is essential to address the specific needs of Kuwait’s diverse population while navigating societal and familial expectations.

## Discussion

This study investigated the perceptions and experiences of mental health clinicians working with children and adolescents in Kuwait, focusing on challenges arising from family, religion, and cultural influences. The findings provide critical insights into the region’s mental health landscape, emphasizing the interplay between cultural dynamics and therapeutic practices. Family serves as a source of emotional support while also acting as a barrier to seeking help due to concerns over family honour and societal expectations (Omu & Reynolds, 2012). These findings align with research highlighting the centrality of family and tribal solidarities in shaping individual behaviors in Kuwaiti society (Al-Ghanim, 2012). Integrating cultural values, such as family involvement, enhances the effectiveness of mental health interventions, particularly for adolescents (Corona et al., 2017).

Stigma, fear of rejection, and mistrust in confidentiality were identified as significant barriers to accessing mental health services, consistent with findings across the Middle East (Eapen & Ghubash, 2004; Khatib et al., 2023). Individuals often avoid professional help due to fears of judgment and damage to reputation. While public attitudes toward mental health are improving in Kuwait, stigma remains pervasive, compounding struggles for children and adolescents requiring familial support (Sewilam et al., 2015). While cultural stigma around mental illness is frequently cited as a barrier to help-seeking in Kuwait, it is important to recognize that such stigma is not unique to this context. Globally, individuals across various societies hesitate to seek mental health care due to fear of being judged, or misunderstood (Maalouf et al., 2019). In Kuwait, this hesitation may be further compounded by systemic challenges, including the limited availability of culturally adapted services, inadequate public mental health education, and a general mistrust of therapy’s effectiveness. Therefore, reluctance to engage in treatment should not be viewed solely as a product of cultural beliefs, but also as a reflection of a mental health system that may not yet fully align with the needs or expectations of the diverse communities it serves.

Religion profoundly shapes attitudes toward mental health, with struggles often perceived as tests of faith or punishments from God. Participants noted that many individuals first seek help from religious advisors, reflecting broader regional trends (Al-Krenawi & Graham, 2000). Stigmatizing beliefs, such as viewing mental illness as caused by supernatural forces or moral failings, contribute to shame and reluctance to seek professional help (Niland, 2022). A gradual shift is emerging toward aligning therapeutic practices with clients’ religious and cultural beliefs, fostering a more inclusive approach to mental health care (Soueif, 2001).

Here, it is important to mention that Kuwaiti society, like many in the Gulf region, is experiencing ongoing cultural shifts as younger generations navigate between traditional values and the influences of globalization. Concepts such as family honour, religious observance, and intergenerational authority continue to play a central role in shaping identity and daily life. However, these values are not static. Increasing exposure to diverse worldviews through education, media, and social interaction has prompted many young people to reinterpret or negotiate these traditions in more individualistic or context-sensitive ways. While some youth may experience tension when traditional expectations conflict with personal or emotional needs, others find ways to integrate both, maintaining respect for cultural heritage while advocating for open conversations around mental health. Similarly, some families are adapting by creating more supportive spaces for dialogue and flexibility. Thus, rather than framing cultural values as fixed obstacles, it is more accurate to view them as dynamic forces that, depending on context, can either hinder or enhance emotional well-being.

Parent coaching was highlighted as a vital therapy component, emphasizing equipping parents with tools to support their children. Participants stressed the need for clear boundaries and collaborative approaches to involve parents in children’s therapeutic journeys. Building trust and addressing cultural sensitivities are essential for fostering a supportive therapeutic environment (Alsaleh, 2012). Raising awareness and reducing stigma were seen as critical for shifting perceptions of mental health. Culturally sensitive practices, combined with educational initiatives, can promote a more accepting societal attitude toward mental health issues (Qureshi et al., 2013). The findings underscore the need for culturally sensitive interventions that respect local values while addressing systemic challenges. Participants highlighted the potential of the ETHICA-4P model to guide ethical reflection, build rapport, and strengthen therapeutic relationships. By incorporating cultural insights, ethical frameworks like ETHICA-4P can help clinicians navigate complexities of mental health care in Kuwait, fostering trust and collaboration.

### Strengths and limitations

This study’s qualitative approach provided rich, contextual insights into Kuwaiti professionals’ mental health perceptions. Given the small sample size, this study does not aim for generalizability but instead seeks to offer in-depth insight into the lived experiences and ethical reflections of clinicians working within Kuwait’s diverse mental health landscape. These themes offer preliminary understanding and suggest areas for future study. Response bias may have also influenced the findings. While client cultural backgrounds were not systematically collected, participants frequently referred to working with Arab clients throughout the interviews. Given the local clinical context, it is likely that most clients shared similar cultural backgrounds. Notably, no participants raised issues related to working cross-culturally, suggesting such cases may have been less frequent or outside the scope of their current caseloads.

The participants’ varying levels of clinical experience and professional training—from early-career counsellors to senior neuropsychologists—may have influenced their ability to navigate cultural nuances in therapy. More experienced therapists, in particular, may have drawn on deeper clinical insight or personal strategies to manage ethical complexities, suggesting a potential link between experience and culturally adaptive practice. Future research could explore how clinical seniority shapes culturally responsive care.

### Future research directions

Future studies should involve larger, more diverse samples to improve generalizability (Maalouf et al., 2019). Longitudinal research could examine how mental health attitudes evolve over time. Exploring interventions, including digital tools and social media, may help reduce stigma. Enhanced training for primary care physicians and culturally sensitive follow-up plans, such as ETHICA-4P, are essential for ethical and effective care (Almazeedi & Alsuwaidan, 2014).

## Conclusion

This study highlights the critical role of cultural, familial, and religious factors in shaping mental health perceptions and practices in Kuwait. Addressing stigma, enhancing community awareness, and adopting culturally sensitive interventions are essential for improving mental health outcomes for children and adolescents. Integrating parental involvement —particularly during the assessment and intervention planning stages—can help tailor therapeutic approaches to the child’s home environment and strengthen family engagement in the healing process. Additionally, leveraging ethical models such as ETHICA-4P can further enhance therapeutic effectiveness while respecting the cultural and religious values of the Kuwaiti context. These insights provide a foundation for future efforts to develop culturally tailored mental health services that align with the unique needs of Kuwaiti society.

## Data Availability

The datasets generated during and/or analyzed during the current study are available from the corresponding author on reasonable request.
